# Effects of High-Intensity Blood Flow Restriction Exercise on Muscle Fatigue

**DOI:** 10.2478/hukin-2014-0044

**Published:** 2014-07-08

**Authors:** Gabriel R. Neto, Heleodório H. Santos, Juliana B. C. Sousa, Adenilson T. A. Júnior, Joamira P. Araújo, Rodrigo R. Aniceto, Maria S. C. Sousa

**Affiliations:** 1 Kinanthropometry and Human Development Laboratory - LABOCINE -UFPB, João Pessoa / PB, Brazil.; 2 Associate Programme on Graduate Program in Physical Education UPE / UFPB, João Pessoa, Paraíba, Brazil.; 3 Federal University of Rio de Janeiro. Physical Education - Graduate Program. Rio de Janeiro, RJ – Brazil.; 4 Graduate Program in Physical Education of University Trás-os-Montes e Alto Douro – UTAD - Vila Real/Portugal.; 5 Laboratory of Kinesiology and Biomechanics - LACIB, Integrated Colleges of Patos, Paraíba, Brazil.

**Keywords:** quadriceps, resistance training, vascular occlusion, athletes

## Abstract

Strength training combined with blood flow restriction (BFR) have been used to improve the levels of muscle adaptation. The aim of this paper was to investigate the acute effect of high intensity squats with and without blood flow restriction on muscular fatigue levels. Twelve athletes (aged 25.95 ± 0.84 years) were randomized into two groups: without Blood Flow Restriction (NFR, n = 6) and With Blood Flow Restriction (WFR, n = 6) that performed a series of free weight squats with 80% 1-RM until concentric failure. The strength of the quadriceps extensors was assessed in a maximum voluntary isometric contraction integrated to signals from the surface electromyogram. The average frequency showed significant reductions in the WFR group for the vastus lateralis and vastus medialis muscles, and intergroup only for the vastus medialis. In conclusion, a set of squats at high intensity with BFR could compromise muscle strength immediately after exercise, however, differences were not significant between groups.

## Introduction

Traditionally, significant increases in strength and muscle mass are expected when the muscles are subjected to loads of moderate to high intensity that correspond to values of at least 70% of 1RM (Campos et al., 2002; McDonagh and Davies, 1984). Thus, it is observed that the overload potentiates the stimuli promoting muscle adaptations and inducing an increase in the recruitment of motor units (MUs) composed of fast twitch fibers, especially in individuals with experience in strength training (ST) (Ahtiainen and Häkkinen, 2009). However, this method seems to result in loss of strength during exercise, once it promotes exhaustion (Fleck and Kraemer, 2004).

In this sense, low intensity ST methods are also capable of improving muscle adaptation levels when combined with blood flow restriction (BFR), by means of external arterial compression (rubber bands or pneumatic tourniquets) during exercise (Loenneke and Pujol, 2009). These improvements are observed in the increased muscle cross-sectional area (Abe et al., 2006) as well as in the capacity of strength production (Takarada et al., 2002), similar to high-intensity ST. However, this ischemic stimulation in the exercise induces a more acidic intramuscular metabolic condition (a decrease in pH by an increase of metabolites), able to produce fatigue that results in failures in the mechanisms of muscle contraction, decreasing the performance during the training session (Cook et al., 2007; Cook et al., 2013).

However, there is a great interest in regulating the force production by the neuromuscular system during exercises, especially in relation to athletes (Ahtiainen and Häkkinen, 2009), as an increase of workload in training sessions is common in training routine, leading an individual to acute fatigue (Häkkinen, 1995). This phenomenon has been identified as a reduction in the ability of force generation leading to work disability (Bigland-Ritchie, 1981). Muscle fatigue may occur due to neurophysiologic mechanisms (Amann and Calbet, 2008). Acute fatigue is usually associated with specific factors of a peripheral source, related to the acidity of the intramuscular environment, causing failures in the neuromuscular junction mechanism (Gandevia, 1992). Thus, a decrease in the amplitude of the electromyography signals during the series of exercise appears to indicate the beginning of fatigue when associated with shortage in the capacity of muscle strength production (Pasquet et al., 2000).

According to Chiu et al. (2004) traditional resistance exercise can result in potentiating and fatiguing responses. A similar fact also occurs with the use of BFR in low intensity counter-resistance exercises (Cook et al., 2007; Pierce et al., 2006). Therefore, there is no evidence in the literature related to the effects of the BFR by external compression in the capacity of force production indicating the appearance of post exercise fatigue during high-intensity exercise series.

Based on previous studies (Cook et al., 2007; Pierce et al., 2006), we set the hypothesis that high-intensity exercise combined with BFR promotes greater impairment in muscle force production immediately after the exercise. Thus, the aim of this study was to investigate the acute effect of high intensity squat on levels of muscular strength production, with and without blood flow restriction.

## Material and Methods

### Participants

Twelve Jiu-Jitsu fighters (age: 25.95 ± 0.84 years, body mass: 80.95 ± 2.75 kg, body height: 173.42 ± 1.18 cm; SBP: 130.50 ± 2.60 mmHg, DBP: 80.0 ± 2.30 mmHg), with minimum experience of two years in strength training participated in the research. Study details and risks were explained to all participants who signed an informed consent form before participating in the research prepared in accordance with the declaration of Helsinki and resolution No. 196/96 of the National Health Counsel. The study protocol was approved by the Research Ethics Committee of the Federal University of Paraíba, (protocol number 0389/11). To be included in the experiment, volunteers had to meet the following criteria: (a) to answer negatively the questionnaire on preventive readiness for physical exercise (PAR-Q); (b) agree not to perform any type of regular physical activity other than the prescribed strength training; (c) be free from any condition that would influence the collection or interpretation of data; and (d) be free from the intake of ergogenic aids that could influence the collection or interpretation of the data.

### Procedures

The study was conducted in the laboratory of the Kinanthropometry and Human Performance at the Federal University of Paraíba. The subjects were randomized into two groups: exercise without restriction of Blood Flow (NFR, n=6) and With Blood Flow Restriction (WFR, n=6). The protocol was performed for eight days, being the first seven days to perform familiarization sessions and moderate physical exercises. The effort intensity was determined two days before, by the 1RM test (Brown and Weir, 2001) in which a load of 80% 1RM was prescribed, characterizing it as a high-intensity exercise (ACSM, 2009). On the eighth day the measurements of isometric strength integrated to surface electromyography signals (sEMG) were conducted. The evaluation of the isometric torque and sEMG was performed before and immediately after the squat series.

Before the exercise session the subjects performed a brief warm up (10 to 15 repetitions at 20% 1RM), followed by an interval of three minutes before the squats series. The range of motion was 180° to 90° of knee extension execution, and the speed was controlled by a metronome (Tagima, Japan) at a rate of 40 rings with 2 beats per minute (1sec/1sec = concentric/eccentric phase). The WFR group performed squats with a twofold 206 latex tube, positioned around the proximal thigh, just below the inguinal region, after pinpointing the blood restriction flow (BRF) with the portable Doppler. The total average of the BRF point was 110 ± 2.35 mmHg, and the percentage determined to compression was 60% of the total restriction in the femoral artery, using approximately 40 seconds (time for pressure setting) before and throughout the series. The compression was released immediately after the exercise. The number of repetitions was recorded by each movement executed in the determined amplitude within the established cadence, and the counting stopped when the subject showed signs of concentric failure, characterized by incomplete execution of the movement or outside the time limit for each contraction stage. The NFR group performed the same procedure but without external compression.

### Torque

The Isometric Torque of the quadriceps extensors was recorded by an integrated system of sEMG-Force (Mendonça, 2000), consisting of a *Bonett* chair with force transducers (Micro-Measurements strain gage, USA) with a gage factor (K) equal to 2.01 and resistance of 350Ω, attached to the arms of the chair. The amplified signal was digitized by an A/D converter board (Lynx with ISA barring) of 16 channels, with 12 bits resolution, using a sampling frequency of 1.000 Hz.

### Surface Electromyography (sEMG)

The software used for the capture and process of the force and the sEMG signals was the (digital polygraph) BioMed application. The surface sEMG signals (Root Mean Square - RMS and Median Frequency - MF) were obtained through active bipolar electrodes (2 cm distance between the positive and negative poles) and synchronized with the system of force measurement, through a biological amplifier (INA 221, *Burr Brown*), with: the ratio of common mode rejection (RCMR) > 95 dB, high input impedance (10 MΩ), low noise (< 5 μV RMS), bandwidth of 10 to 490 Hz, and a gain of 1500 times. The electromyographic signals captured were converted to the digital format with a sampling rate of 1,000 samples/s and the RMS was normalized for each subject by the peak signal (% peak sEMG) at each contraction (Fraga et al., 2008; Knutson et al., 1994).

For data recording, the subjects were sitting with the knee flexed at 60° (Kulig et al., 1984) and the ankle fixed to the resistance arm of the system force, the torso and pelvis fixed by bands in order to avoid compensatory movements. Then, the subjects were asked to perform three maximal voluntary isometric contractions (MVIC) for five seconds with 60 second intervals between them (Davies et al., 2000).

For the calculation of strength, the first and last second of each MVIC was ignored, considering the maximum value obtained in three seconds and the largest of the three MVIC was used as a reference for strength. The quadriceps muscle activation was measured by the *Root Mean Square* (RMS) magnitude of the rectus femoris (RF), vastus lateralis (VL) and vastus medialis (VM) and the median frequency (MF) of the respective muscles. The whole essential process for sEMG signal capture occurred in accordance with the *Surface Electromyography for the Non Invasive Assessment of Muscles* (SENIAM) recommendations (Hermens et al., 2000).

### Statistical Analysis

All data showed a normal distribution by the Shapiro-Wilk test and homogeneity of variances (p > 0.05), and were expressed as mean and standard error (SE). Two-Way ANOVA [group (WFR vs NFR) × time (Pre vs Posttest)] followed by the Bonferroni post hoc test was used for all dependent variables. The percentage of changes in the dependent variables was calculated by the equation: Δ% = (post × pre-exercise) / pre-exercise × 100. All analyses were performed with Statistical Package for Social Sciences (SPSS 18.0 for Windows) and the level of significance was set at p < 0.05.

## Results

### Isometric Torque

**Figure 1:**
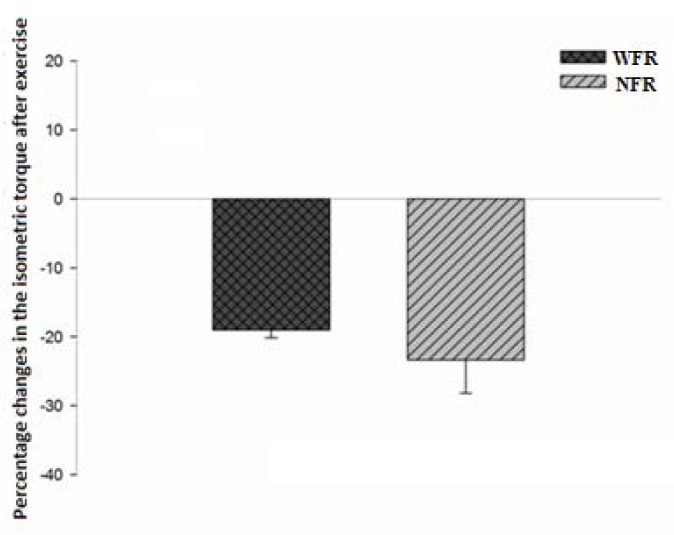
Percentage changes in maximum isometric torque (pre × post-exercise) for WFR and NFR groups NFR = without Blood Flow Restriction; WFR = With Blood Flow Restriction Average values and standard error

Isometric torque values were significantly reduced during a MVIC immediately after exercise when compared to the pre-exercise condition (Table 1), both for NFR (p = 0.001) and WFR (p = 0.014) groups. It was found in the intergroup analysis (Figure 1), that negative percentage changes that occurred in isometric torque as a result of the series of exercise were similar in NFR and WFR (p > 0.05).

### Median Frequency

**Figure 2:**
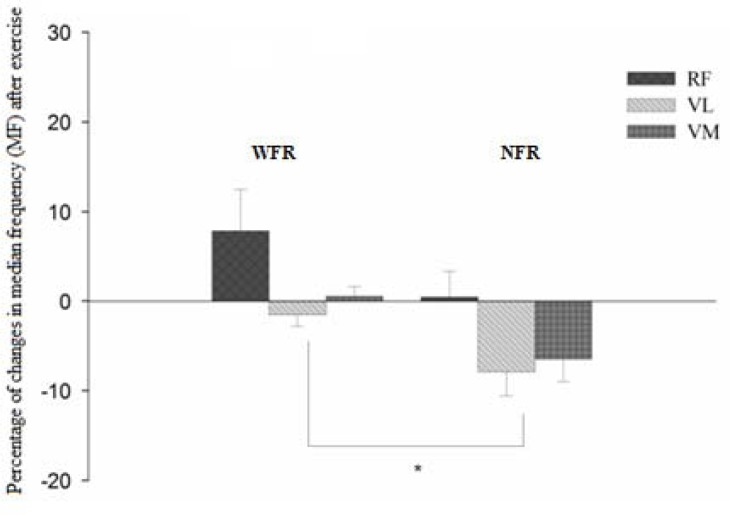
Percentage of changes in median frequency (MF) in a MVIC for knee′s three extensor muscles Average values and standard error ^*^Significant difference between groups (p < 0.05); Average values and standard error, NFR = without Blood Flow Restriction; WFR = With Blood Flow Restriction; RF - Rectus femoris; VL - Vastus lateralis; VM - Vastus Medialis

The median frequency showed no significant changes compared to the pre-exercise condition for the NFR group (Table 1). However, the WFR group showed a significant MF reduction in the in VL (p = 0.032) and VM (p = 0.048) muscles. In intergroup comparison (Figure 2), the percentage changes were similar for BRF and VL muscle (p > 0.05), but in the WFR group, the VM muscle showed greater percentage reduction (p = 0.030).

### sEMG Amplitude

**Figure 3:**
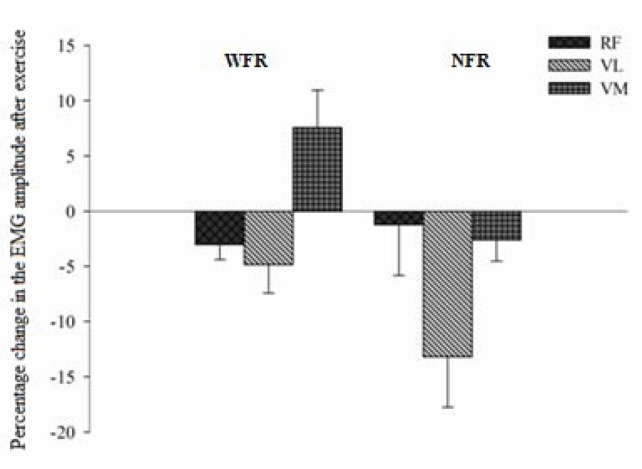
Percentage change in the sEMG amplitude in a MVIC for knee′s three extensor muscles NFR = without Blood Flow Restriction; WFR = With Blood Flow Restriction; RF - Rectus femoris; VL - Vastus lateralis; VM - Vastus Medialis; Average values and standard error

The sEMG signal was normalized by the peak during a MVIC. During the intergroup analysis, both conditions (WFR and NFR) showed no significant changes in sEMG amplitude during the course of a MVIC right after the squats series. The grade of muscle activation of the RF, VL and VM of subjects who underwent effort in the NFR group did not change in comparison to the pre-exercise condition (p > 0.05); in the WFR group, only VL showed significant reduction in signal activation (p = 0.049), as shown in Table 1. In intergroup comparison (Figure 3), changes in the sEMG amplitude signal were not significant (p > 0.05).

## Discussion

The present study investigated the acute effect of high intensity squat on levels of muscular fatigue, with and without blood flow restriction. Thus, the results demonstrate the acute onset of neuromuscular fatigue after a number of exercise (squat) until concentric failure with the use of high loading (80% 1RM), typically used to increase muscle strength. Studies relate that high intensity strength training sessions induce significant reduction in muscle strength as well as changes in the neuromuscular system responsible for exercising muscles (Häkkinen, 1995). In this experiment, a series of high-intensity exercise, characterized by concentric-eccentric contractions induced loss of ability to generate strength, represented by reductions in isometric torque in both groups NFR (−19%) and WFR(−23,3%). As already reported, the decrease in muscle strength after intense exercise can be characterized by an acute onset of muscle fatigue (Bigland-Ritchie, 1981). The study by Cook et al. (2007) determined the effect of eight BFR protocols on muscle fatigue (decrement in maximal voluntary contraction [MVC] after the performance of exercise), and compared the decrement in MVC with the currently recommended resistance exercise intensity (∼80% MVC). The authors concluded that all BFR protocols elicited at least as much fatigue as high-load, even though lower loads were used. The 20% protocol was the only one that elicited significantly more fatigue than high-load. It seems that performing exercises with and without the restriction of blood flow at high loads shows similar responses when performed with lower loads (20% MVC), which was observed by the reduction of isometric torque in the present study.

The information concerning the above mentioned percentage reduction in force seems to be in line with recent findings in the literature. The study carried out by Walker et al. (2012) in active individuals evaluating neuromuscular fatigue in two exercise protocols with different adaptive traits (strength and hypertrophy) in relation to the specific exercise to increase strength showed a decrease in maximal isometric torque in knee extensors (approx. −30%), accompanied by sEMG amplitude reduction of −12% (VL′s and VM′s mean). Likewise, Ahtiainen and Hakkinen (2009) conducted a study using high intensity exercise with athletes vs. non-athletes to evaluate neural fatigue and muscle activation after two exercises protocols (maximum and forced repetitions). They observed a decrease of approximately 69% in isometric torque (also knee extensors) in athletes when exposed to conditions of forced repetitions, but this difference did not seem substantial in relation to non-athletes. In relation to muscle activation (sEMG) of BFR, VL and VM, authors also found no significant difference between the groups. The differences found in relation to the torque values between the present study and the investigations conducted by Ahtiainen and Häkkinen (2009) and Walker et al. (2012) can be assigned to variables inherent to the exercise (load, number of repetitions, series and blood flow restriction condition), since the present study showed a low effort volume in a single series.

The present study observed an influence of higher loads on force changes. This fact partially explains the reduction in isometric torque by using loads of predetermined intensity to near maximal effort (1RM), implying more recruitment of motor units composed of fast twitch fibers (type II), which are directly associated with the muscle strength production (Ahtiainen and Häkkinen, 2009).

In the present investigation, during MVICs of knee extensors performed post-exercise, there were no significant changes in the sEMG amplitude of the BFR and VM, there was only a significant reduction in VL (−3.6%) in the WFR group. Studies using sEMG to evaluate neuromuscular fatigue reported that the decrease in the amplitude signal is associated with the beginning of fatigue through central processes that involve impairments in nerve conduction (Karabulut et al., 2010). This process of central fatigue is related to changes in efferent motor command, influenced by an increase in H^+^ and P_i_ concentrations, implying the inhibition of *α* motor neurons as well as a decline in nerve conduction to the supraspinatus muscle (Babault et al., 2006).

It is possible that because of the use of external compression device (twofold latex tubes) during the exercise, the individuals of the WFR group have been led to an ischemic stimulus related to reduced oxygen availability (Amann and Calbet, 2008) and metabolites accumulation (H^+^ e P_i_ ions). In this sense, the study by Wernbom et al. (2009) corroborates our findings. The authors conducted a study that investigated muscle activity and endurance during fatiguing low-intensity dynamic knee extension exercise with and without BFR. They concluded that BFR during low-intensity dynamic knee extension decreases endurance but does not increase the maximum muscle activity compared with training without restriction when both regimes are performed until failure. It appears that a series of squats and three sets of unilateral knee extensions performed by WFR and NFR groups show no differences in EMG activity.

In relation to changes in MF, significant reductions in the force spectrum of the VL and VM were observed (−18.5% and −18.2%, respectively) after a set of squats in the WFR group. A decrease in MF was identified in a study that evaluated fatigue induced by high-intensity exercise (Babault et al., 2006) and differed from the results obtained in other (Karabulut et al., 2010); these discrepancies are attributed to issues related to the methodology used in these experiments, once the muscle activation signals were measured under dynamic exercises with different protocols.

The reduction in MF may be associated with the synchronization of motor units (Bigland-Ritchie et al., 1981) and with the impairment in conduction velocity to the fibers, due to higher concentrations of H^+^ changing pH values (Place et al., 2010). Thus, the authors speculate that changes in the acidity of the intramuscular environment would imply losses in sensitivity / reduction of Ca^2+^ from the sarcoplasmic reticulum, resulting in impaired contractile function during strength production (Place et al., 2009). Therefore, due to limitations inherent to the method used in this research, it was not possible to analyze such variables to indicate peripheral fatigue, attributing the detriments in MF to possible central mechanisms, assuming central fatigue (Vestergaard-Poulsen et al., 1995). Regarding the comparison groups (WFR vs. NFR), the VM responded differently from the others in the blood flow restriction condition exercise, showing a significant percentage change (signal decrease) related to the VM in the group without flow restriction. A similar fact was observed by Pierce et al. (2006), where the MF of the VM resulted in significant changes in the group exercise under ischemic conditions.

However, different answers in muscle activation can be observed through the exercise type (mono or multi-joint), so the squat series until concentric failure may have led to reductions in sEMG signals, not expected in exercise involving a single joint (Ahtiainen et al., 2003). Findings of the present study complement available data on high intensity strength exercises combined with blood flow restriction in relation to the long-term neuromuscular adaptations, since the study by Laurentino et al. (2008) using the technique of blood flow restriction with training of the knee extensors, in a period of eight weeks, did not find significant neuromuscular adaptations in relation to the high-intensity condition without blood flow restriction. Therefore, the results of this study should be interpreted with caution, because of methodological limitations related to the external pressure control (blood flow restriction), quantity of exercises (just one series) and the absence of biochemical markers analysis (eg.: Lactate).

Thus, we suggest that further studies with different protocols for high intensity exercise with blood flow restriction in athletes are made in order to elucidate factors that imply greater neuromuscular fatigue, seeking better planning in prescription and training periodization.

## Conclusion

It may be concluded that a set of high intensity squats with BFR can compromise muscle strength immediately after the exercise, as observed in the reduced median frequency in VM and VL muscles. However, high intensity exercise with BFR does not seem to compromise muscle performance, when compared to the same exercise without BFR.

## Figures and Tables

**Table 1:** Values obtained in the pre vs post-exercise and between groups for the variable workload

Variable/Group	NFR (n=6)		WFR (n=6)	

	Pre	Post	Pre	Post
Isometric Torque (Kgf)	92.97±1.99	75.35±2.47^*^	93.13±7.37	69.88±3.53^*^
F_med_ RF (Hz)	152.66±8.81	163.73±8.53	160.55±4.71	161.13±5.44
F_med_ VL (Hz)	167.96±5.32	165.36±5.24	157.22±4.41	145.18±7.52^*^
F_med_ VM (Hz)	175.46±16.75	175.78±15.31	137.04±6.43	128.57±8.36^*^
RF (%Peak_sEMG_)	21.30±0.01	20.62±0.01	21.64±0.01	21.34±0.01
VL (%Peak_sEMG_)	18.15±0.01	17.10±0.10	24.80±0.20	21.23±0.15^*^
VM(%Peak_sEMG_)	13,54±0,04	15.16±0.33	21.55±0.13	21.00±0.11

* Significant difference (p < 0.05) compared to the pre-exercise (intragroup).

Kgf - Kilogram Force; Hz - Hertz;% Peak_sEMG_ - Standardization thought the sEMG signal peak; NFR = without Blood Flow Restriction; WFR = With Blood Flow Restriction; RF - Rectus femoris, VL - Vastus lateralis; VM - Vastus Medialis
